# An efficient and extensible approach for compressing phylogenetic trees

**DOI:** 10.1186/1471-2105-12-S10-S16

**Published:** 2011-10-18

**Authors:** Suzanne J Matthews, Tiffani L Williams

**Affiliations:** 1 Department of Computer Science and Engineering, Texas A&M University, College Station, Texas, USA

## Abstract

**Background:**

Biologists require new algorithms to efficiently compress and store their large collections of phylogenetic trees. Our previous work showed that TreeZip is a promising approach for compressing phylogenetic trees. In this paper, we extend our TreeZip algorithm by handling trees with weighted branches. Furthermore, by using the compressed TreeZip file as input, we have designed an extensible decompressor that can extract subcollections of trees, compute majority and strict consensus trees, and merge tree collections using set operations such as union, intersection, and set difference.

**Results:**

On unweighted phylogenetic trees, TreeZip is able to compress Newick files in excess of 98%. On weighted phylogenetic trees, TreeZip is able to compress a Newick file by at least 73%. TreeZip can be combined with 7zip with little overhead, allowing space savings in excess of 99% (unweighted) and 92%(weighted). Unlike TreeZip, 7zip is not immune to branch rotations, and performs worse as the level of variability in the Newick string representation increases. Finally, since the TreeZip compressed text (TRZ) file contains all the semantic information in a collection of trees, we can easily filter and decompress a subset of trees of interest (such as the set of unique trees), or build the resulting consensus tree in a matter of seconds. We also show the ease of which set operations can be performed on TRZ files, at speeds quicker than those performed on Newick or 7zip compressed Newick files, and without loss of space savings.

**Conclusions:**

TreeZip is an efficient approach for compressing large collections of phylogenetic trees. The semantic and compact nature of the TRZ file allow it to be operated upon directly and quickly, without a need to decompress the original Newick file. We believe that TreeZip will be vital for compressing and archiving trees in the biological community.

## Background

**Figure 1:**
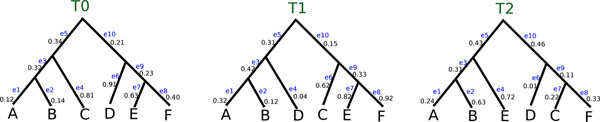
**Example trees.** A collection of three evolutionary trees on six taxa labeled *A* to *F.* Each edge *e_i_* represents an evolutionary relationship (or bipartition) along with a value that represents the length of the branch.

In a phylogenetic tree, living organisms occupy the leaves and ancestral organisms are internal nodes, with the edges of the tree denoting evolutionary relationships (see Figure 1). The task of phylogenetics is to infer this tree from observations (e.g., molecular sequences) obtained from existing organisms of interest. To reconstruct a phylogenetic tree, the most popular techniques (such as MrBayes [1] and TNT [2]) often return tens to hundreds of thousands of trees that represent equally-plausible or closely-related hypotheses (or candidate trees) for how the taxa evolved from a common ancestor. Given that phylogenetic searches return tens to hundreds of thousands of candidate evolutionary trees, biologists need new techniques for managing and sharing these large tree collections effectively. As biologists obtain more data to produce evolutionary trees, phylogenetic techniques must reconstruct larger trees, resulting in ever-larger collections of candidate trees. Thus, there is a critical need to develop phylogenetic compression techniques that reduce the requirements of storing large tree collections so that they can be shared easily with colleagues around the world.

**Figure 2:**
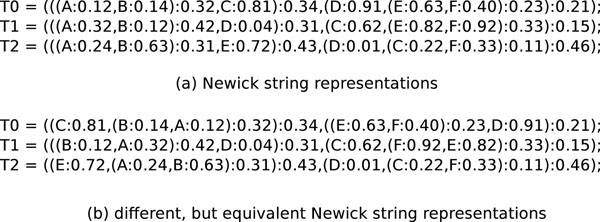
**Example Newick string representations.** Newick representations for the phylogenetic trees shown in Figure 1. Two different, but equivalent, Newick representations are given for each tree.

We introduced TreeZip [3], a novel compression algorithm that reduces the requirements over standard compression algorithms (such as 7zip) for storing and sharing large collections of evolutionary trees. Given that many of the evolutionary relationships in a collection of phylogenetic trees are shared, the novelty of the TreeZip approach is storing such relationships only once in the compressed representation. TreeZip compresses a Newick file based on the semantic representation (i.e., tree bipartitions and unique topologies). The Newick format [4] is the most widely used file format to represent phylogenetic trees. In this format, the topology of the evolutionary tree is represented using a notation based on balanced parentheses (see Figure 2). A Newick formatted tree uses nested parentheses to represent the evolutionary relationships (or subtrees) within a phylogenetic tree. In a Newick file, each tree is located individually on separate lines. Figure 2 shows two sets of different, but equivalent Newick representations for the three trees shown in Figure 1. Matching pairs of parentheses symbolize internal nodes in the evolutionary tree.

However, the Newick representation of a tree is not unique. For a particular tree with *n* taxa, there are *O*(2*^n^*^–1^) different (but equivalent) Newick strings to represent its topology. Consequently, general-purpose data compression techniques cannot leverage domain-specific information regarding the Newick file. Thus, our TreeZip approach shows that there is great potential for obtaining good compression by utilizing the semantic information in a Newick file of evolutionary trees.

### Related work

Besides TreeZip, the only other known phylogenetic tree compressor known by us is the Texas Analysis of Symbolic Phylogenetic Information (TASPI) algorithm [5,6]. The benefits of TASPI include compressing phylogenetic trees and computing their consensus. TreeZip shares these benefits as well as the ability to handle branch lengths, merge tree collections, and extract subsets of trees directly from the compressed TreeZip file. Also, the authors of TASPI state that their approach is not robust to the *O*(2*^n^*^–1^) different Newick representations of a phylogenetic tree. Our experimental results show that TreeZip's performance is not impacted by these different Newick representations.

### Our contributions

In this paper, we improve upon our TreeZip algorithm in three significant ways. First, we extend TreeZip to handle weighted phylogenetic trees containing branch lengths as shown in Figure 1. Next, we show the extensibility of the TreeZip compressed format when given a Newick file representing a collection of *t* trees. That is, in addition to extracting all of the trees contained in a compressed TRZ file, we show how the TreeZip format can be used to perform additional extraction operations (such as returning the set of unique trees) and constructing majority and strict consensus trees. Our final extension shows how we can use set operations (such as union, intersection and set difference) on TRZ files to merge tree collections. We experimentally study the performance of our TreeZip algorithm in comparison to 7zip on four biological data sets, including freshwater (20,000 trees over 150 taxa), angiosperms (33,306 trees over 567 taxa), fish (90,002 trees over 264 taxa) and insects (150,000 trees over 525 taxa) tree collections. Of these datasets, the first three are weighted (have branch lengths), while the last is unweighted. Our largest (smallest) tree collection consists of 150,000 (20,000) trees requiring 434 MB (67 MB) of storage space. However, due to the storage requirements of weighted tree collections, our fish dataset consisting of 90,002 264-taxa trees has the largest file size of 533 MB.

Overall, our results show that the compressed TreeZip (TRZ) file is over 74% smaller than the original Newick file on weighted collections. On unweighted collections, it is 98% smaller. When TreeZip is coupled with 7zip, the resulting TRZ+7zip file is on average 92% smaller on weighted collections. On unweighted collections, the TRZ+7zip file is in excess of 99.8% smaller than the original Newick file. Given that there are *O*(2*^n^*^–1^) different Newick representations for a phylogenetic tree, we study the impact of these different, but equivalent representations on both the TreeZip and 7zip approaches. The results show that as the number of different Newick representations increases, there is a significant increase in 7zip's compressed representation. TreeZip, on the other hand, is robust to changes in the Newick string representation of a tree. Furthermore, not only does TreeZip produce a smaller compressed file than 7zip, it often does so in a time that is faster or comparable to 7zip.

Beyond decompressing a TRZ file to its original Newick representation, our experiments provide exciting results related to the flexibility of extracting additional information from the compressed file. Of interest to biologists are the unique set of trees that are contained in their tree collection (or compressed TreeZip file). Moreover, we can output the strict and majority consensus trees from the phylogenetic data in the TRZ file in less than one second on the tree collections studied in this paper. Since the TRZ file is text, various set operations can be quickly and efficiently performed on the TRZ representation of a weighted (unweighted) collection of trees up to 5 (60) times faster than on the Newick representation. Thus, our results show that the TRZ file is an effective and extensible compressed format that biologists can leverage to manage their large tree collections.

### Paper structure

The rest of this paper is organized as follows. In our Methods section, we describe the TreeZip algorithm, including the mechanisms behind compression, decompression, and our set operations functionality. We also describe our experimental methodology. We describe and discuss our experimental results in our Results and Discussion section. Lastly, we summarize our findings in Conclusions.

## Methods

The TreeZip algorithm is composed of two main parts: compression and decompression. In the subsections that follow, we first discuss the process of compression, in which a Newick input file is transformed into the TreeZip compressed format, or TRZ file. Next, we discuss decompression, in which a TRZ file is used to reconstruct the desired set of phylogenetic trees in Newick format. We note here that since any phylogenetic tree with *n* taxa has *O*(2*^n^*^–1^) equivalent Newick string representations, any one of these equivalent Newick string representations can be used as the decompressed version. We continue with a description of the algorithm behind the TreeZip set operations. Unlike the compression and decompression functions, the TreeZip set operations take as input two TRZ files, and outputs a single TRZ file. In this manner, set operations are performed in the context of a TRZ file, without any loss of space savings. Lastly, we present a summary of our experimental methodology.

### Compression

In the Newick input file, each string *i*, which represents tree *T_i_*, is read and stored in a tree data structure. During the depth-first traversal of input tree *T_i_*, each of its bipartitions is fed through two universal hash functions, *h*_1_ and *h*_2_[7]. There are 2(*n* – 1) total bipartitions contained in each tree *T_i_*, where *n* is the number of taxa. Thus, each of the 6-taxa trees in Figure 1 contains 10 bipartitions. Both of the universal hashing functions require as input a *n*-bit bitstring representation of each bipartition in tree *T_i_*. Taxa are ordered lexicographically, where *b*_0_ represents the first bit and the first taxon name in the ordering, *b*_1_ is the second bit representing the taxon in the ordering, etc. For the phylogenetic trees shown in Figure 1, the taxa ordering is *A*, *B*, *C*, *D*, *E*, and *F.* The bitstring 100000 represents the bipartition *A*|*BCDEF*, which corresponds to edge *e*_1_ in each of the three trees. In tree *T*_0_, edge *e*_5_ corresponds to the bipartition *ABC*|*DEF* or bitstring 111000, which is formed by performing an OR operation on the bitstrings of its children represented by edges *e*_3_ and *e*_4_. The bipartition *DEF*|*ABC* corresponds to the bitstring 000111, which has a branch length of 0.21 denoted by edge *e*_10_ in tree *T*_0_.

#### Step 1: storing bipartitions in a hash table

The hash function *h*_1_ is used to generate the location (index) for storing a bipartition in the hash table. *h*_2_ is responsible for creating a unique and short bipartition identifier (BID) for the bipartition so that the entire *n*-bit bitstring does not have to be analyzed in order to insert bipartitions into the hash table. Our two universal hash functions are defined as follows:  and . *R* = (*r*_0_, …, *r_n_*_–1_) is a list of random numbers in the range of (0, …, *m*_1_ – 1), and *S* = (*s*_0_, …, *s_n_*_–1_) is a list of random integers between (0, …, *m*_2_ – 1). *B* = (*b*_0_, …, *b_n_*_–1_) is a bipartition represented as an *n*-bit bitstring. *m*_1_ represents the number of entries (or locations) in the hash table. *m*_2_ represents the largest bipartition ID (BID) given to a bipartition. *b_i_* represents the *i*th bit of the *n*-bit bitstring representation of the bipartition *B.*

**Figure 3:**
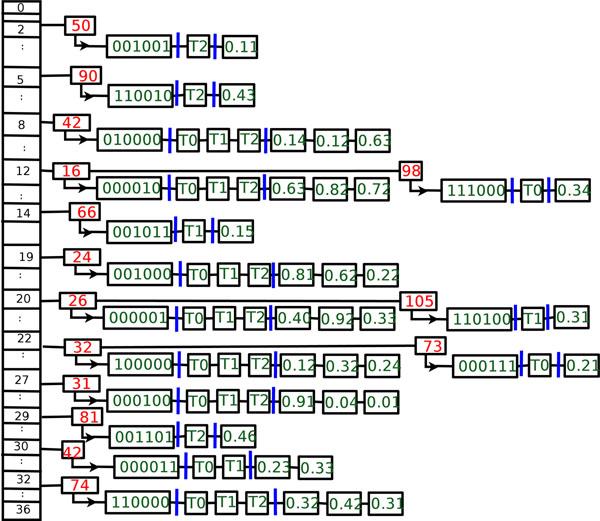
**Internal hash table.** Our hash table data structure for the phylogenetic trees shown in Figure 1.

Figure 3 shows how the bipartitions from Figure 1 are stored in our hash table. Each entry represents a unique bipartition and the hash line consists of a BID, its bitstring representation, a list of trees that contain that contain it, and the respective branch lengths for each of trees. In this figure, *R* = (22, 45, 19, 27, 12, 20), *S* = (32, 42, 24, 31, 16, 26), *m*_1_ = 37, and *m*_2_ = 3, 701.

The first three lines of the compressed TRZ file represent the taxa names, the number of trees in the file, and the number of unique bipartitions. Afterwards, we process each hash table row which will represent a line in the compressed file. There are three components (bitstrings, tree ids, branch lengths) to a TRZ line. We also note here that bipartitions stored in the TRZ file are stored in sorted order according to the number of ones they contain. Ties are broken lexicographically. This guarantees that if two tree collections have equivalent one-to-one corresponding sets of trees, the TRZ files of the two collections will be *identical* despite differences in the Newick string representations. Below, we describe how TreeZip encodes each of these components.

#### Step 2: encoding bitstrings

Once all of the bipartitions are organized in the hash table, we begin the process of writing the TRZ compressed file, which is a plain text file. We run-length encode our bitstrings. Run-length encoding is a form of data compression in which runs of data (i.e, sequences in which the same data value occurs in many consecutive data elements) are stored as a single data value and count, rather than as the original run. For the bitstring 110000 in Figure 3, we would have a run-length encoding of 1:2⌴0:4, where each *x* : *y* element represents the data value (*x*) and the number of repetitions (*y*)*.* The ⌴ character denotes a space. Since bitstrings can either contain runs of 1s or 0s, we introduce two new symbols. 1: is encoded as K, while 0: encoded as L. (We use characters A through J for compressing our list of tree ids described shortly.) Hence, we encode the bitstring 110000 as K2L4. In our experiments, we considered taking every group of 7 bits in our bitstring and translating it to an ASCII character. However, we were able to get better compression by using run-length encoding, which showed significant benefits on our biological tree collections consisting of hundreds of taxa.

#### Step 3: identifying and encoding the set of unique tree ids

Let  represent the set of evolutionary trees of interest, where *.* For a bipartition *B*,  represents the set of the trees in  that share that bipartition.  is the set of trees that do not share bipartition *B.* Since these sets are complements, their union comprises the set *.* To minimize the amount of information present in our TRZ output, we print out the contents of the smaller of these two sets. If , then we output . Otherwise,  is outputted. In our TRZ file, we denote  and  lines with the '+'and '-' symbol, respectively.

Even with use of the smaller of the  or  sets, the list of tree ids can get very large. This is due to the fact that as *t* grows large, the number of bytes necessary to store a single id also grows. We note first that a tree *T* can be represented as a *k*-bit bitstring, where *k* is the number of bipartitions discovered in the collection. If we feed these *k*-bit bitstring representations into a slightly modified version of the above hash functions, we can obtain the set of unique trees, *U*, where |*U*| = *u*. This set of unique trees are given the corresponding tree ids of 0…*u* – 1, and will represent the total set of trees in consideration with any bipartition. Duplicate information is encoded and stored at the end of the TRZ file.

Since the trees are inserted into the hash table in their order of appearance in the Newick file, our lists of tree ids will be in increasing order. As a result, we store the differences between adjacent elements in our tree id list. These differences are then run-length encoded. To eliminate the need for spaces between the run-length encoded differences, the first digit of every element is encoded as a character, with 0…9 represented by A…J. Consider bitstring 000011, which is in row 30 (its *h*_1_ value) in our hash table shown in Figure 3 and has an *h*_2_ value of 42. The  set will be used for this bipartition, and its run-length encoded differences will be 2, which will be encoded as C. Given the large number of shared bipartitions in a collection of trees that result from a phylogenetic search, there will be many more unique trees than unique bipartitions. Hence, encoding the differences in the tree ids leverages the sharing among the trees—especially since 1 is the most common difference between adjacent elements in the tree id lists.

#### Step 4: encoding branch lengths

The last item to process on a hash line are the branch lengths associated with a unique bipartition. Branch lengths take the form *x.y*, where *x* is the integral and *y* the mantissa. For this domain, branch lengths tend to be very small (*x* = 0). Hence, we use this property to our advantage by only encoding the integral in special cases (*x* > 0). For these special cases, we store the integral separately along with its related tree id. On the datasets studied here, at least 99.6% of the branch lengths begin with 0. The mantissa corresponds to a fixed number, *k*, of digits. For our tree collections, *k* = 6. To encode the mantissa, we take two digits at a time (since we can guarantee this value fits into a byte) and translate it into a readable ASCII character. For example if we have a value of 99 as input, we add 33 to create the corresponding Extended ASCII readable character ä. It is necessary to add 33 to any input value since the first 32 characters in ASCII are non-printable, control characters. We tried different universal integer encodings (e.g., variable byte encoding) [8], but given the range of integers represented by *k* digits, the various integer encodings did not result in a smaller compressed file. This is due to the fact that when *k* = 6, universal codes become less effective than straight binary as the size of the integers themselves increase [8]. Furthermore, we achieve better compression by feeding the resulting TRZ file to a general-purpose compressor such as 7zip.

However, when using variable byte encoding for the branch lengths, 7zip's algorithm could not reduce the file size any further and resulted in a much larger compressed file.

Lastly, we note that branch lengths are not compacted like tree ids since the branch lengths originate from an infinite set of real numbers. Tree ids, on the other hand, are drawn from a finite set of *t* tree ids ranging from 0 to *t* – 1.

### Decompression

The two major steps of the decompression in TreeZip are decoding the contents in the TRZ file and rebuilding the collection of *t* trees. Decoding reconstructs the original hash table information which consists of bipartition bitstrings and the tree ids that contain them. When the TRZ file is decoded, each line of the file is processed sequentially. First, the taxa information is fed into TreeZip. Next, the number of trees is read. Each bipartition is then read sequentially.

#### Decompression data structures

To assist in bipartition collection, we maintain two data structures *M* and *N*, both which are *t* × *k* matrices, where *k* = 2*n* is the maximum number of bipartitions for a phylogenetic tree. The length of each matrix corresponds to the number of trees specified in the TRZ file. Each row *i* in matrix *M* corresponds to the bipartitions required to rebuild tree *T_i_*. The corresponding row in matrix *N* is the list of associated branch lengths. For example, in Figure 3, the bipartition at row 32 of our hash table (it's *h*_1_ value) is shared among all the trees. It is therefore added to every row in *M.* To *N*, we add the value 0.32 to *N*[0], 0.42 to *N*[1] and 0.31 to *N*[2], signifying that these are the associated branch lengths for the corresponding bipartition in *M.* On the other hand, the bipartitions 11100 and 001001 are contained only in trees *T*_0_ and *T*_2_ respectively, and therefore will be added to *M*[0] and *M*[2]. Thus, we also add branch lengths 0.34 and 0.11 to *N*[0] and *N*[2]*.* Since each bipartition is processed in order in our TRZ file, we are able to guarantee a one to one correspondence between the values in *M* and *N.* We also maintain a separate data structure that stores duplicate tree information to assist in the construction for *M* and *N.*

#### Flexible decompression

Decoded bitstrings are the basic units for building trees. Once the bitstrings, associated tree ids and branch lengths are decoded, we can build the original trees one by one. In order to build tree *T_i_*, the tree building function receives as input matrix row *M*[*i*] which contains the bipartitions encoded as bitstrings for tree *T_i_*, and matrix row *N*[*i*] which contain the associated branch lengths for each bipartition in *M*[*i*]*.* Each of the *t* trees is built starting from tree *T*_0_ and ending with tree *T_t_*_–1_, whose bipartitions (branch lengths) are stored in *M*[0] (*N*[0]) and *M*[*t* – 1] (*N*[*t* – 1]), respectively. The trees are reconstructed in the same order that they were in the original Newick file. However, given *O*(2*^n^*^–1^) possible Newick strings for a tree *T_i_*, the Newick representation that TreeZip outputs for tree *T_i_* will probably differ from the Newick string in the original file. This is not a problem semantically since the different strings represent the same tree.

To build tree *T_i_*, it is initially represented as a star tree on *n* taxa. A star tree is an bitstring representation consisting of all 1's. In the TRZ file, bipartitions are stored in decreasing order of their bitstrings. This means the when it is time to rebuild trees, the bipartitions that group together the most taxa appear first. The bipartition that groups together the fewest taxa appears last in the sorted list of '1' bit counts. For each bipartition *i*, a new internal node in tree *T_i_* is created using the bitstring in *M*[*i*], and the associated weight is added using the value in *N*[*i*]*.* Hence, the taxa indicated by the '1' bits become children of the new internal node. The above process repeats until all bipartitions are added to tree *T_i_*.

The decompressor can also output sub-collections of trees of interest to the user. For example, if the user was interested in the set of unique trees in the collection (rather than the entire collection), TreeZip can return this set of trees of interest to the user. In addition, TreeZip has built-in functionality to return the strict and majority-rule consensus trees of an encoded collection of trees in a couple of seconds to the user. The strict and majority-rule consensus trees are especially of interest to biologists, since this is the summary tree structure that commonly appears in publications. Furthermore, these subcollections of trees can be produced directly from the TRZ file, without a need to decompress the original collection. In other words, operations can be performed directly on the TRZ file without requiring a loss of space savings. This is not the case with standard compression approaches which produce unreadable binary output. In these cases, the original file must always be fully decompressed in order for any operations to be performed, resulting in zero space savings.

### Set operations

One of our goals is to show that the TRZ file represents a viable alternative archive format to the Newick file for representing large collections of trees. If the same set of operations can be performed on a TRZ file that can be done on a Newick file, then we can argue that the two file types are equivalent. In order to accomplish this goal, we implemented a series of set operations that exploits the textual structure of the TRZ file to produce sets of trees of semantic interest. The set operation functions in TreeZip takes as input two TRZ files and outputs a single TRZ file that represents the results of a particular set operation. Here, we implement three set operations in total: *union*, *intersection*, and *set difference.* The union between two collections of trees is defined as the set of unique trees that exist over both collections. The intersection between two collections is defined as the set of unique trees that exist in the first collection and the second collection. The set difference between two collections is defined as the set of unique trees that exist in the first collection and not in the second. For example, consider the collections stored in Files 1 and 2 in Table 1. In this example, each file contains three trees for a total of six trees labeled from 1 to 6. Trees 1 and 4 are identical. All other trees are distinct from each other. The union between the trees in files 1 and 2 consists of five trees (trees 1, 2, 3, 5, and 6). The intersection consists of one tree (tree 1), and the set difference consists of two trees (trees 2 and 3).

**Table 1:** Two Sample Files of Weighted Trees

File 1	
1.	(((A : 0.12, B : 0.13) : 0.14, C : 0.15) : 0.16, (D : 0.17, (E : 0.18, F : 0.19) : 0.20) : 0.21);
2.	(((A : 0.11, B : 0.34) : 0.29, D : 0.23) : 0.22, (C : 0.24, (E : 0.25, F : 0.26) : 0.27) : 0.28);
3.	(((A : 0.29, B : 0.11) : 0.31, E : 0.33) : 0.15, (D : 0.38, (C : 0.36, F : 0.37) : 0.32) : 0.31);

File 2	

4.	(((E : 0.18, F : 0.19) : 0.20, D : 0.17) : 0.21, (C : 0.15, (A : 0.12, B : 0.13) : 0.14) : 0.16);
5.	(((A : 0.34, B : 0.23) : 0.21, C : 0.53) : 0.24, (F : 0.41, (E : 0.13, D : 0.51) : 0.21) : 0.33);
6.	(((A : 0.12, B; 0.43) : 0.21, C : 0.06) : 0.20, (E : 0.04, (D : 0.28, F : 0.33) : 0.02) : 0.41);

TreeZip is able to perform these set operations (and other operations) quickly since the set of unique bipartitions and trees are known and already encoded into the TRZ file. TreeZip then uses this encoded information to create a new TRZ file with the set of desired trees without needing to rebuild the tree structures. On the other hand, if one were to attempt to perform these operations on a Newick file, the bipartitions from each tree will have to be extracted and the relationships between the set of trees will have to be discovered every single time. As tree collections grow large, this can pose a significant overhead. Lastly, we stress again that these set operations can all be performed on the input TRZ files without any loss of space savings. This is of critical interest, as it shows the viability of using the TRZ file as an alternative format for storing trees. With standard compression methods, the resulting binary file must always be decompressed in order for any type of manipulation on the data to be performed. As a result, these could not be considered as alternative formats to the Newick file. The TRZ file on the other hand is a viable format because set operations can be performed on it. Furthermore, since there is no loss of space savings, the TRZ file is a more efficient way of storing collections of trees.

### Experimental methodology

Our implementation of TreeZip used in the following experiments can be found at http://treezip.googlecode.com. Experiments were conducted on a 2.5 Ghz Intel Core 2 quad-core machine with 4 GB of RAM running Ubuntu Linux 8.10. TreeZip is written in C++ and compiled with gcc 4.4.3 with the -02 compiler option.

#### Biological trees

Below, we provide a description of the four biological tree collections used in this study. Our tree collections include trees with weighted and unweighted branches. While more details are provided in the references for our published tree collections, weighted trees were obtained by running a Bayesian phylogenetic analysis using MrBayes [1]. The unweighted trees were derived from a maximum parsimony analysis using TNT [2]. For each dataset, the Newick tree file contains *t* trees in the input file. All of the weighted collections we use for our experiments contain binary trees. The unweighted insects dataset, however, contains multifurcating (or non-binary) trees.

1. freshwater: 20,000 weighted trees obtained from an analysis of 150 taxa (23 desert taxa and 127 others from freshwater, marine, and oil habitats) [9]. The size of the Newick file for this tree collection is 67 MB. There are 1,318 unique bipartitions out of 5, 960, 000 total bipartitions.

2. angiosperms: 33,306 weighted trees obtained from an analysis of a 567 taxa (560 angiosperms, seven outgroups) [10]. The size of the Newick file for this tree collection is 429 MB. There are 3,011 unique bipartitions out of 37, 702, 392 total bipartitions.

3. fish: 90,002 weighted trees obtained from an analysis 264 fish taxa (an unpublished collection from M. Glasner's lab at Texas A&M University). Only binary trees are contained in this dataset. The size of the Newick file for this tree collection is 533 MB. There are 12,379 unique bipartitions out of 47, 341, 052 total bipartitions.

4. insects: 150,000 unweighted trees obtained from an analysis 525 insect taxa [11]. The trees contained in this set are multifurcating. The size of the Newick file for this tree collection is 434 MB. There are 573 unique bipartitions out of 157, 200, 000 total bipartitions.

#### Measuring performance

We compare TreeZip to the 7zip compression algorithm. In our previous work [3], we found that 7zip is the most effective method for compressing phylogenetic trees amongst the standard compression methods (e.g. gzip, bz2). We measure the performance of our TreeZip algorithm in two primary ways: space savings and by using different, but equivalent Newick strings. Please note that 7zip here represents a Newick file compressed with the 7zip compression scheme.

##### Space savings and running time

We use the *space savings* measure to evaluate the performance of TreeZip in comparison to general-purpose compression algorithms. The space savings *S* is calculated as . A higher space savings percentage denotes better compression of the original file. The goal is to get the level of space savings as close to 100% as possible. A value of 0% indicates no difference from the uncompressed, original Newick file. We also use running time to calculate how long each algorithm requires to compress and decompress a file. Time is shown in seconds.

##### Different, but equivalent Newick representations

As mentioned previously, for any given tree of *n* taxa, there are *O*(2*^n^*^–1^) Newick string representations associated with it. Since general purpose compression methods such as 7zip compress tree files by looking for redundancy at the Newick string level, they are unable to efficiently compress trees when there is a lack of redundancy in the Newick string representations. To illustrate this, we created a different, but equivalent Newick file for each dataset. For a Newick file containing *t* trees, each tree receives a different, but equivalent Newick representation. We note that using different, but equivalent Newick representations does not change the size of the resulting Newick file. For example, our fish dataset consisting of 90,002 trees over 264 taxa requires 533 MB of storage space. The Newick file containing different, but equivalent Newick strings still occupies 533 MB of disk space.

## Results and discussion

In this section, we explore the compression and decompression performance of 7zip, TreeZip, and TreeZip+7zip. Our previous results [3] show that 7zip is the best general-purpose compressor in comparison to gzip and bzip. The TreeZip+7zip compressed format is the TreeZip (TRZ) format which is then fed to 7zip for further compression. Moreover, our previous study showed that TreeZip outperforms TASPI. Since no implementation of TASPI is available and since none of the trees we had available that were used in the TASPI experiments had branch lengths, we could not compare TreeZip to TASPI in the context of this study.

Finally, each point in the plots represents the average performance over three runs.

### Compression performance

**Figure 4:**
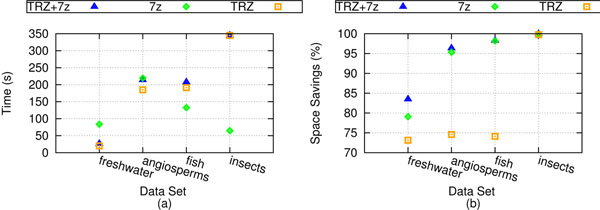
**Compression performance.** Compression performance for our biological datasets. In this figure, (a) shows running time of compression approaches, while (b) shows space savings.

Figure 4 shows the performance of TreeZip's compression algorithm. Figure 4(a) shows run-time information, and Figure 4(b) shows space savings results. On the freshwater and angiosperms datasets, TreeZip is faster than 7zip. However, as the number of trees under consideration increases in size, so does the amount of time needed for compression. In terms of size, the TRZ file by itself is larger than the 7zip file. However, we obtain an average of 75% space savings on our weighted collections, and about 99% space savings on our unweighted collection. The discrepancy in space savings between the weighted and unweighted cases underlines the complexity of compressing branch lengths. However, we note that when the TRZ file is combined with 7zip, the TRZ+7zip file has space savings on average of about 96%. 7zip by itself, on the other hand, averages about 93% space savings.

**Figure 5:**
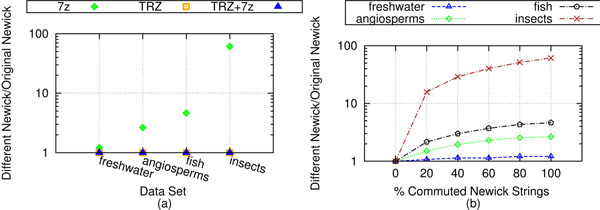
**Compression performance on different, but equivalent Newick strings.** Compression performance for our biological datasets using different, but equivalent Newick strings. (a) Unlike TreeZip and TreeZip+7zip, 7zip experiences an increase in compressed file size when different, but equivalent Newick strings are introduced (100% commuted). TreeZip and TreeZip+7zip experience no change. (b) A closer look at how the percent of different, but equivalent Newick strings affect the increase in file size of 7zip (*p*% commuted). As more Newick strings are randomly commuted, the performance of 7zip becomes increasingly worse.

To measure the effects of branch rotations on our datasets, we took each set of trees and gave them a random, but equivalent Newick string representation. We refer to this process as commuting the Newick representation. Figure 5 shows the performance of the various compression schemes on different, but equivalent Newick string representations. The TRZ and TRZ+7zip files did not increase in file size. 7zip took up to 4.4 times longer on this new file. Figure 5(a) shows the change in space savings of the different compression schemes between the equivalent Newick files and the original files. Here, 100% of the Newick strings in the file have been commuted. A value of 1 signifies no change in file size. The space savings achieved by TreeZip and TreeZip+7zip does not change, despite the use of different, but equivalent Newick strings. This highlights TreeZip's robustness to branch rotations. This is not the case for 7zip. On our weighted sets (freshwater, angiosperms, fish), the size of the 7zip compressed file became almost 4 times larger. On the unweighted set (insects), the 7zip compressed file becomes 61 times larger. This is equivalent of an increase of the size of the 7zip compressed Newick file from 696 KB to 38 MB.

Figure 5(b) highlights the increased compressed file sizes obtained by 7zip on different, but equivalent Newick string representations. The x-axis indicates the percent of the original file that received commuted Newick string representations. For each percentile, *p* percent of the trees in the file contain a different, but equivalent Newick string representation. The 0% mark is the original Newick file. All the datasets have a universal value of 1 at this point, since there is no change in the compression quality. The 100% mark is equivalent to the files that were used in Figure 5(a). As the number of Newick strings that are randomly commuted increases, 7zip has a corresponding decrease in compressed file size performance. While TreeZip is slower than 7zip in terms of execution speed, robustness to branch rotations provides TreeZip with a significant advantage over general-purpose compressors such as 7zip.

### Decompression performance

**Figure 6:**
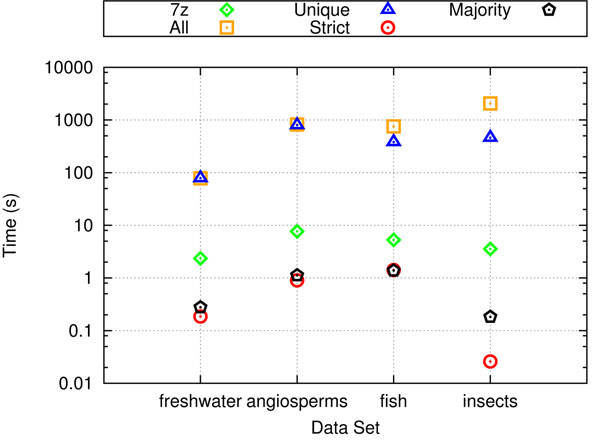
**Decompression performance.** Decompression performance for our biological datasets.

Figure 6 shows the decompression performance of 7zip and TreeZip-based decompressors. When decompressing all of the trees in the compressed file to their original Newick representation, 7zip is a faster decompressor than the TreeZip-based approaches. For our two largest datasets (angiosperms and fish), 7zip is two orders of magnitude faster than TreeZip and TreeZip+7zip. However, a major advantage of TreeZip is that its decompression algorithm is flexible. TreeZip can return all of the trees that are contained in a compressed file (similarly to 7zip), but it can also return other types of output such as the set of unique trees, the strict consensus tree, and the majority consensus tree. Consensus trees plays a major role in summarizing a phylogenetic analysis and having such an operation that can work directly on compressed trees is an added advantage. Furthermore, these operations can be executed quickly in TreeZip. For example, on the datasets studied here, strict and majority consensus trees can be produced in less than second, which is significantly faster than current consensus tree algorithms such as HashCS [12] that work directly from a Newick tree file. Since the 7zip file is binary, none of these operations can be performed easily on its compressed representation. In order to find the set of unique trees in a 7zip file, this file would have to be decompressed to the original Newick tree file followed by executing a separate procedure to determine the unique trees based on their Newick representations.

### Set operations performance

Next, we evaluate the performance results of set operations on Newick files, Newick files compressed with 7zip, TRZ files, and TRZ files compressed with 7zip. Each of our four datasets consists of *r* runs of trees. That is, for the freshwater dataset, two runs of MrBayes was required to generate the 20,000 trees in the collection. For the remaining datasets, *r* = 12 for the angiosperms trees, *r* = 2 for the fish trees, and *r* = 5 for the insects trees. Runs are labeled from *R*_0_…*R_r_*_–1_.

To create a single data sample for the set operation experiments, we randomly create a bitstring *B* of length *r*, where a 1 in location *B_i_* states that trees from run *R_i_* should be used in the set operation experiments and a 0 means that trees from that run will not be used. Using the bitstring *B* as a guide, we create a vector *S* that contains the identities of those runs that will participate in the set operations experiment. For example, if *B* = 01011, then *S*_0_, *S*_1_ and *S*_2_ would contain runs *R*_1_, *R*_3_ and *R*_4_, respectively. We randomly generate a set operation (union, intersection, or set difference) to apply to the trees represented by *S*_0_ and *S*_1_*.* Let *U* represent the result. Next, we take the result *U* and apply a random set operation to it using trees from *S*_2_. We continue in this manner until |*S*| – 1 set operations have been applied randomly. The set operations and the order in which they are applied are also recorded. For each of our four datasets, the above procedure is repeated 100 times in order to create 100 data samples. Furthermore, for a particular dataset, all set operation experiments applied to the Newick, Newick+7zip, TRZ and TRZ+7zip files use the same 100 data samples along with the same ordering of how the set operations are applied to the data. Our plots show the average running times and file sizes over these 100 data samples.

**Figure 7:**
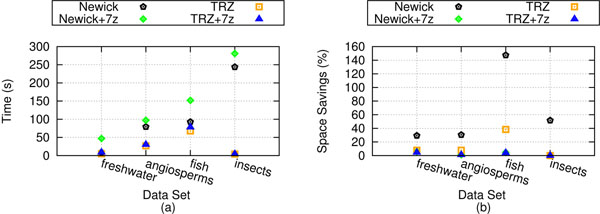
**Set operations performance.** Performance of set operations on our biological datasets. (a) The running time of a random collection of set operations run on different file formats. (b) The amount of disk space required by the result of the set operations.

Figure 7 shows our performance results of set operations performed on Newick files, Newick files compressed with 7zip, TRZ files, and TRZ files compressed with 7zip. Figure 7(a) shows running time results. On weighted trees, it is up to 3 times faster to perform set operations on TRZ files over Newick files. On the unweighted case, it is about 55 times faster. While there is little overhead in combining the TRZ file with 7zip, there is more significant overhead when combining Newick files with 7zip. While it is only at most 10 seconds slower to combine 7zip with a TRZ file for set operations, the overhead of combining 7zip with a Newick file is as much as a minute on our experimental platform. As a result, the speedup results are more significant when comparing set operations on a TRZ+7zip file versus a Newick+7zip file. On weighted trees, the speedup is at most 5.25. On unweighted trees, the speedup is up to 62.4. The differences in speedup between the unweighted and weighted tree sets is related to the extra processing required by TreeZip to handle the weights on the tree branches.

Figure 7(b) shows the average space savings of storing the results of set operation in a Newick file, a TRZ file, Newick+7zip file, and a TRZ+7zip file. In terms of size, the results of set operations are more efficiently stored in TRZ files than Newick files. On weighted trees, the TRZ file storing the results of the set operations is 74.1% smaller than the Newick file. On the unweighted case, it is up to 99.7% smaller. This is very consistent with the general space savings of using a TRZ file over a Newick file on weighted and unweighted trees respectively. TRZ+7zip files have at most a 21% improvement in space savings over Newick+7zip files in the weighted case. On the unweighted case, the TRZ+7zip takes up 79% less space than the Newick+7zip file. Together, these results demonstrate the benefit of using the TRZ file for performing set operations.

## Conclusions

There is a critical need for phylogenetic compression techniques that reduce the space requirements of large tree collections. In order to reconstruct the true tree, phylogenetic searches can easily return tens of thousands to hundreds of thousands of candidate evolutionary trees for biologists to consider. To help biologists handle these large collections of trees, we extend our previous TreeZip algorithm [3] in several significant ways. First, the TreeZip algorithm is augmented to allow for the compression of trees with weighted branches. Second, we offer an extensible decompressor which allows for filtering and extraction of sets of trees of interest. Lastly, TreeZip can perform fast set operations directly on its compressed TRZ file.

Our experimental evaluation of TreeZip shows that it compresses a Newick file into a plain text TRZ representation that is at least 73% smaller than the original file on weighted trees and over 98% smaller on unweighted trees. When combined with 7zip, the TreeZip+7zip file achieves an average space savings of 92% on the weighted case, and a space savings of over 99% on the unweighted case. Our results also show that TreeZip's performance is robust to different Newick representations of the same phylogenetic tree. The space savings achieved by 7zip, on the other hand, decreases as the number of different Newick representations for the same phylogenetic tree increases.

However, TreeZip's most powerful advantage arises from its flexible compressed file format. Since the TRZ file is in plain text, we can easily design extensible decompressors that extract the relevant phylogenetic tree information of interest. In this paper, we illustrate two decompression applications (identifying the unique set of trees in a file and computing consensus trees) that can extract information quickly from a TRZ file. Furthermore, we showed how we can leverage the TRZ format to design set operations (union, intersection, set difference) to merge tree collections of interest. Our study showed that set operations can be performed up to five times faster on a TRZ file than on a Newick file. Furthermore, the set operation results occupy up to 99% less space in a TRZ file as compared to its Newick counterpart.

Overall, our results show that TreeZip can play a vital role in helping biologists manage their large phylogenetic tree collections effectively. Our future work includes augmenting the extensible decompressor with additional applications and optimizing our implementation to improve TreeZip's running time. We also plan to explore how to extend TreeZip for use beyond phylogenetic trees.

## Authors' contributions

SM designed and implemented the TreeZip algorithm, performed all of the experiments, and created all of the figures. TW also designed the TreeZip algorithm along with its experimental evaluation. Both authors contributed to writing the manuscript and have approved its final contents.

## Competing interests

The authors declare that they have no competing interests.
